# The ghrelin and leptin responses to short-term starvation vs a carbohydrate-free diet in men with type 2 diabetes; a controlled, cross-over design study

**DOI:** 10.1186/s12986-016-0106-x

**Published:** 2016-07-22

**Authors:** Frank Quentin Nuttall, Rami Mahmoud Almokayyad, Mary Carol Gannon

**Affiliations:** Section of Endocrinology, Metabolism & Nutrition, and the Metabolic Research Laboratory (111G), Minneapolis VA Health Care System, 1 Veterans Drive, Minneapolis, MN 55417 USA; Department of Medicine, Division of Diabetes, Endocrinology and Metabolism, University of Minnesota, 516 Delaware Street, SE, MMC 101, Minneapolis, MN 55455 USA; Present address: Park Nicollet Health Care System, Minneapolis, MN USA; Department of Food Science & Nutrition, University of Minnesota, 1334 Eckles Avenue, St Paul, MN 55108 USA

**Keywords:** Ghrelin, Leptin, Carbohydrate-free diet, Type 2 diabetes, Dietary carbohydrates, Dietary fats, 24-hour profile

## Abstract

**Background:**

We recently have reported the 24-hour glucose, insulin and glucagon responses to a 72-hour fast compared to a 72-hour macronutrient-sufficient, carbohydrate-free diet in men with type 2 diabetes. The 72-hour time period was used because it is the time required for the major metabolic adjustments to a lack of food to be instituted. As part of that study, ghrelin and leptin responses were monitored.

**Methods:**

Twenty-four-hour total ghrelin and overnight fasting leptin concentrations were determined in males with type 2 diabetes when ingesting a standard, mixed meal diet (control), followed by a carbohydrate-free diet for 72 h or were starved for 72 h, using a crossover design.

**Results:**

A rise in ghrelin concentration before and a decrease after meals was present when the standard diet was ingested. However, in contrast to literature reports in normal subjects, a circadian variation was not apparent. Meal related changes were absent with starvation. A carbohydrate-free diet resulted in a daylong decrease in ghrelin. It also resulted in a 19 % decrease in the overnight fasting leptin concentration. Leptin was decreased 54 % with total starvation.

**Conclusion:**

Ingestion of a typical mixed-meal diet results in meal-related changes in ghrelin similar to those reported in normal subjects, although the circadian rhythm was not apparent. Except for the lack of meal-related changes, starvation did not change the concentration. A carbohydrate-free, high fat diet resulted in a daylong suppression of ghrelin. The leptin concentration was decreased by both the carbohydrate-free diet and starvation.

**Trial registration:**

ClinicalTrials.gov Identifier: NCT01469104.

## Background

We recently have reported the 24-hour glucose, insulin and glucagon responses to a 72-hour fast compared to a 72-hour macronutrient-sufficient, no-carbohydrate diet in men with type 2 diabetes [1]. The 72-hour time period was used because it is the time required for the major metabolic adjustments to a lack of food to be instituted [2, 3]. It also is a time period over which a loss in non-water body mass is minimal [3]. Our overall objective was to compare the results when the metabolic fuel being oxidized is largely fat, with or without a deficiency of an exogenous fuel supply. In addition, dietary carbohydrate elimination has been reported to mimic, in many regards, the metabolic response to short-term starvation (fasting). A remarkable decrease in the 24-hour integrated glucose and insulin concentrations occurred (49 % and 68 %, respectively). Approximately 70 % of the glucose response and 72 % of the insulin response to short-term starvation could be attributed to removal of carbohydrate from the diet.

As part of that study, ghrelin and leptin responses were monitored. The results are presented and discussed in the current report. We were particularly interested in determining these effectors since there is a paucity of data in the literature regarding their response to dietary manipulations in people with type 2 diabetes. Although not well characterized, ghrelin and leptin are considered to be important in fuel metabolism.

## Methods

### Study aim, design and setting

The aim of the study was to determine the effect of a carbohydrate-free (CHO-free) diet and starvation on the 24-hour profile of total ghrelin concentration in subjects with type 2 diabetes.

This was a crossover study design with a four-week washout period between study arms. On one occasion the subjects starved, on the other occasion they received a calorie-sufficient, CHO-free diet for 72 h. The order in which subjects were assigned to receive a CHO-free diet or were starved was alternated. Each subject was provided with a standardized dinner (55 % carbohydrate, 15 % protein, and 30 % fat), based on 25 kcal/kg body weight, to be ingested at home at 1800 h the day before the admission day. After that, only water was allowed until admission to the Special Diagnostic & Treatment Unit (SDTU, similar to a Clinical Research Center) the next morning.

For the first 24 h of each study arm the subjects ingested a standard diet consisting of 55 % carbohydrate, 30 % fat, 15 % protein. For the next 72 h they either starved, or ingested a CHO-free diet consisting of <3 % carbohydrate, 15 % protein, ~ 85 % fat. The CHO-free diet contained the same amount of protein as the standard diet. Dietary fat replaced the carbohydrate.

**Fig. 1:**
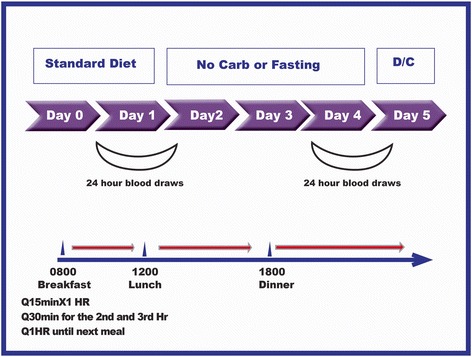
Study Design. A standard diet consisting of 55 % carbohydrate, 15 % protein, and 30 % fat was ingested for dinner at home at 1800 h before admission, and during day 1 in the SDTU. On one occasion during days 2–4 subjects starved, on the other occasion they ingested a carbohydrate-free diet. Meals were ingested at 0800, 1200 and 1800 h. During two 24.5 h periods, from 0730 day 1 to 0800 day 2, and again from 0730 day 4 to 0800 day 5, blood was drawn a total of 42 times (3 baseline, every 15 min for 1 h after a mealtime, every 30 min for the 2nd and 3rd hours after a mealtime, and then every hour until the next mealtime

On the day of admission, each subject reported to the SDTU at 0700 h. An indwelling IV catheter was inserted on that day, and blood samples were obtained at 0730, 0745 and 0800 h for baseline determinations. Subjects received the standard breakfast, lunch and dinner at 0800, 1200 and 1800 h, respectively. Blood samples were obtained every 15 min after each meal for the first hour, every 30 min for the second and third hour and hourly after that until the next meal or until 0800 h of the next morning (Fig. 1).

On the second day, subjects were asked to starve for 72 h, or were provided with the CHO-free meals at 0800 h, 1200 h and 1800 h for 72 h. The total food energy distribution was breakfast 32 %, lunch 40 %, dinner 28 %. The subjects consumed all of the food provided without difficulty.

Ingestion of water was encouraged. Black coffee, tea without sugar or cream, and calorie-free beverages were allowed. The subjects were confined to the SDTU and were under nearly constant surveillance. Activity was limited to quiet diversions such as reading or watching TV. Smoking was not allowed during the study. Blood samples were obtained during last 24 h of the 72-hour intervention, with the same time intervals as the first day. Thus, the data presented for the CHO-free arm or starved arm represent the responses from the last 48 through 72 h of the study (Fig. 1).

With this design, each arm of the study had its own mixed meal control. Thus, we compared each of the CHO-free and starvation data sets with their own respective standardized mixed meal control diets when analyzing the data.

### Participants

Seven male subjects with untreated type 2 diabetes were studied in a clinical research unit (SDTU). All subjects met the American Diabetes Association criteria for the diagnosis of type 2 diabetes [4]. One subject was untreated; 3 subjects had been receiving metformin; 3 subjects had been receiving glipizide. These medications were discontinued for 24 or more days before the study, after obtaining approval by the subjects primary care providers. Thyroid, renal and liver function tests were normal. Subject characteristics have been published previously [1]. Briefly, the mean age was 60 years (range 49 – 72); mean weight was 97 ± 6 kg (range 81 – 130); mean BMI 31 ± 2 kg/m^2^ (range 25 – 38).

The 24-hour ghrelin data were incomplete for one subject when ingesting the CHO-free diet. Therefore those data, and his data when ingesting the corresponding control diet, were not used in the analysis of results, i.e. the results are for n = 6. For the 6 subjects, the mean age was 60 years (range 49 – 72); mean weight was 100 ± 7 kg (range 88 – 130); mean BMI 31 ± 2 kg/m^2^ (range 25 – 38).

Written informed consent was obtained from all subjects, and the study was approved by the Department of Veterans Affairs Medical Center Internal Review Board (IRB).

### Assays

Total ghrelin was assayed using radioimmunoassay (RIA) kits manufactured by Linco (St. Louis, MO); leptin was assayed using an enzyme-linked immunosorbent assay (ELISA) kit manufactured by R & D Systems (Minneapolis, MN).

### Area determination

The net integrated 24-hour area responses were calculated using the overnight fasting concentration as baseline with a computer program based on the trapezoid rule [5].

### Statistics

Statistics were determined by using paired Student’s *t*-test with Prism Software (Graphpad Software, Inc. San Diego, CA) for the iMac computer (Apple, Cupertino, CA). A p-value less than 0.05 was the criterion for significance. Data are presented as the mean ± SEM. Individual data points were not statistically evaluated for the 24-hour ghrelin profiles.

## Results

### Total ghrelin

#### Carbohydrate (CHO)-free diet (Fig. 2)

**Fig. 2:**
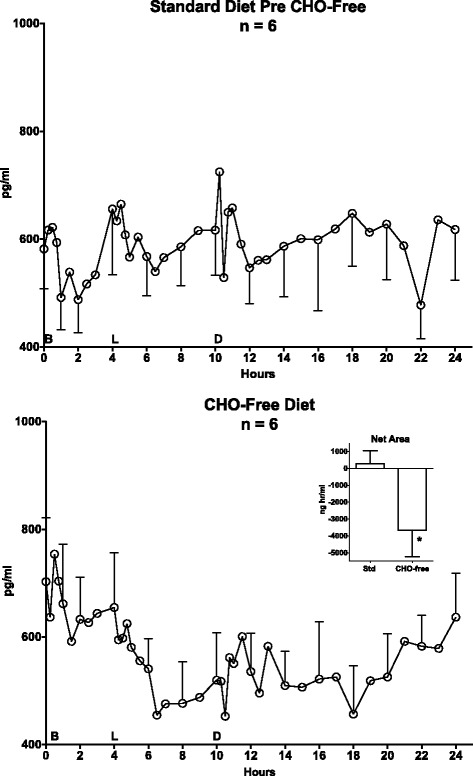
Carbohydrate-Free Diet 24-hour Total Ghrelin Response. Twenty-four hour mean total ghrelin responses in 6 men with type 2 diabetes while ingesting a standard diet (*top*) and during the last 24 h of a 3-day a carbohydrate-free diet (*bottom*). B, L, D on the x-axis indicate the time of breakfast, lunch, and dinner. Insert: Control (standard) Diet (286 ± 765 pg^.^hr/ml) and the CHO-free Diet (-3666 ± 1575 pg^.^hr/ml) 24 h Ghrelin Net Areas. The differences in area responses are statistically significantly different (*P* = 0.04)

The overnight fasting total ghrelin concentrations at the beginning of the 24-hour collection period were 582 ± 74 and 703 ± 119 pg/ml for the control (top) and CHO-free days (bottom), respectively.

While ingesting the control diet, there was a decrease in ghrelin concentration after the breakfast meal. It then increased prior to the lunch meal and subsequently decreased, but the decrease was less than following the first meal. The concentration increased before the dinner meal and then decreased and remained stable overnight. Qualitatively, a circadian change in baseline was not apparent (Fig. 2, top).

When ingesting the CHO-free diet the total ghrelin concentration decreased modestly until the midday meal after which it rapidly decreased further, reaching a nadir 6.5 h after breakfast. Except for a modest transient increase after the midday meal, it remained depressed and was stable until 21 h, when it returned to the previous overnight fasting value (Fig. 2, bottom). That is, a negative circadian rhythm was observed.

#### Net area responses

When ingesting the control diet, the 24-hour total ghrelin *net area* response, using the overnight fasting value as baseline, was essentially zero (286 ± 765 pg^.^hr/ml), i.e. unchanged from baseline. However, it was considerably decreased when the subjects ingested the CHO-free diet (-3666 ± 1575 pg^.^hr/ml) (*P* = 0.04) (Fig. 2 bottom, insert).

#### Short-term starvation (Fig. 3)

**Fig. 3:**
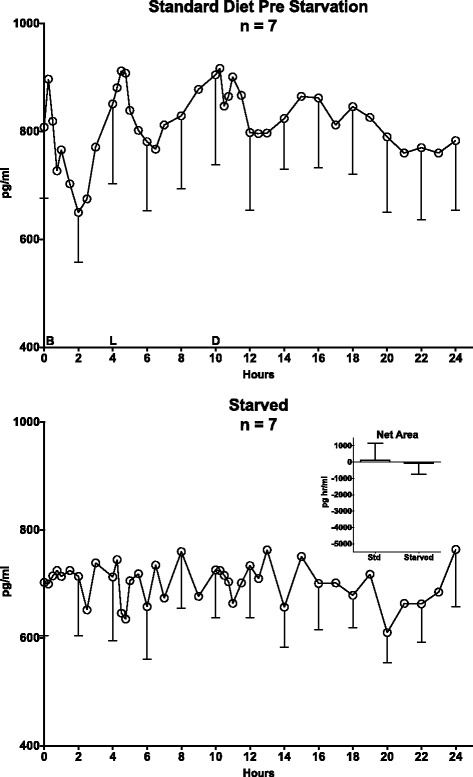
Starvation 24-Hour Total Ghrelin and Insulin Responses. Twenty-four hour mean total ghrelin responses in 7 men with type 2 diabetes while ingesting a standard diet (*top*) and during the last 24 h of a 3-day fast (*bottom*). Insert: Control (standard) Diet (118 ± 1042 pg^.^hr/ml) and Starvation (-56 ± 678 pg^.^hr/ml) 24 h Ghrelin Net Areas. The differences in area responses were not statistically different (*P* = 0.72)

The overnight fasting total ghrelin concentrations at the beginning of the 24-hour profile data collection period were 808 ± 131 and 703 ± 100 pg/ml for the control (top) and starvation days (bottom), respectively (*n* = 7).

With starvation, the concentration remained essentially stable throughout the 24-hour period of study (Fig. 3, bottom). An intrinsic variation associated with meals was not present, that is the pre-post meal oscillations noted with the control diet were not observed. The control diet responses (Fig. 3 top) were similar to those obtained with the control diet for the CHO-free arm (Fig. 2 top). Again, a circadian change was not apparent either with food ingestion or with starvation.

#### Net area responses

The 24-hour total ghrelin net area responses, using the overnight fasting values as baseline, were similar following the ingestion of the control diet (118 ± 1042 pg^.^hr/ml) or during the last 24 h of starvation (−56 ± 678 pg hr/ml) (*P* = 0.89), (Fig. 3 bottom, insert).

In summary, when ingesting the standard diets, the pre and post meal oscillations were present. The net 24-hour integrated ghrelin area values were unchanged when compared to the overnight fasting value. The area also was unchanged with starvation. Only for the CHO-free diet was it different, i.e. it was decreased.

### Overnight fasting leptin concentrations (Table 1)

**Table 1:** Leptin data (*n* = 7)

Plasma/Serum	Control for CHO-Free	CHO-Free	Control for Starved	Starved
Leptin (ng/ml)	10.0 ± 3.2	8.1 ± 2.7*	11.4 ± 4.9	5.3 ± 1.8*

*P < 0.05 Student’s *t* test

Following ingestion of the CHO-free diet, the overnight fasting leptin concentration decreased from 10.0 to 8.1 ng/ml (*P* = 0.01). With starvation it decreased from 11.4 to 5.3 ng/ml (*P* = 0.04). Thus, with elimination of carbohydrate from the diet the decrease was 19 %. After the period of starvation it was decreased by 54 %.

## Discussion

### Ghrelin

Several years ago, the 24 h circulating total ghrelin concentration was determined in normal subjects (9 females, 1 male) when typical (45 % CHO, 35 % fat, 20 % protein) mixed meals were ingested throughout the day [6]. The ghrelin concentration decreased episodically and rapidly after each meal and increased prior to the next meal. In addition, an underlying circadian rhythm was reported to be present with a nadir at 0900 h and an acrophase late at 0100 h [6].

The data therefore, suggested that rapid, food-induced changes in ghrelin could be regulating the dynamics of feeding and fasting throughout the day. The authors also commented that the circadian rhythm was similar to that of leptin, which also had been implicated in fuel regulation.

The same investigator group [7] later again determined the 24-hour total ghrelin profiles in a longer-term study in which ordinary foods were ingested. Normal subjects (16 females and 2 males) were provided a weight-stable diet (45 % CHO, 35 % fat, 20 % protein) for 2 weeks. The ghrelin 24-hr profile then was determined. Clearly defined decreases after meals, with rises before meals, were present. This again was on a background of rising concentrations during the day. That is, a well-defined and reproducible circadian rhythm was reported.

When the diet was changed to a high carbohydrate diet (65 % CHO, 15 % fat, 20 % protein) the profiles were similar [7]. The meal-related excursions were modestly greater when the high carbohydrate meals were ingested, but the 24 h integrated profile and area responses were reported to be unchanged. In the same study, a weight-loss regimen utilizing a high carbohydrate diet also did not result in a change in the 24-hr profiles or the integrated areas under the curves. The background circadian rhythm did not change regardless of the changes in diet.

In another study [8] the authors reported 24 h total ghrelin profiles in lean subjects and in obese subjects (8 females and 5 males) before and after a diet-induced weight loss. The subjects ingested regular mixed meals (55 % CHO, 30 % fat, 15 % protein). In obese subjects the profiles were similar, but the overnight fasting ghrelin concentration was lower, when compared to the lean subjects. Overall the meal-related changes were similar but somewhat less prominent [8].

In a previous 5-week study, we determined for the first time, the total 24-hour ghrelin profiles in subjects with untreated type 2 diabetes ingesting typical mixed meals (55 % CHO, 30 %fat, 15 % protein) [9]. The expected rise in ghrelin before meals and a post-meal decrease was present. The responses were similar to those reported in normal subjects, but not as well defined. In addition a circadian rhythm noted in normal subjects [7, 10] was not apparent [9]. The 24-hour background concentrations were stable and little changed from the initial fasting value. In the present study, the control diet was similar to that used in our previous study. As expected, the results were similar (Figs. 2 and 3).

Whether the lack of a background of rising concentrations during the day (circadian rhythm) in the present and previous study is unique to subjects with diabetes remains to be determined. It cannot be attributed merely to obesity [11]. Of interest, Norrelund et al. [12] reported that somatostatin strongly lowered ghrelin concentrations. Thus it may be playing a role in ghrelin regulation and may be modified in people with type 2 diabetes.

In our previous study [9] changing the macronutrient composition from 55 % CHO, 30 % fat, 15 % protein to 30 % CHO, 40 % fat, and 30 % protein had little effect on the ghrelin profiles. Thus, changes in macronutrient composition typically obtained in mixed meals had little effect on the meal-related changes in ghrelin response in people without [7] or with type 2 diabetes.

The effect of individual macronutrients remains somewhat unclear. In several single meal studies, carbohydrate (particularly glucose) ingestion resulted in a rapid decrease in ghrelin [13–19]. Thus dietary carbohydrates, if ingested independently or largely independently, and in sufficient amounts, can rapidly lower the ghrelin concentration in normal subjects. Ingested protein in one study did not result in a decrease in ghrelin [20]; in another it resulted in an increase [18]. Ingested lipids also had no effect on the ghrelin concentration in men and only had a very modest lowering effect in women [20]. Others reported it only weakly lowered it [16], had no effect [19], or actually increased it [18].

Later, Foster-Schubert et al. [10] reported that when ingested as 80 % of the test macronutrient, dietary carbohydrate (absorbed glucose), protein or fats ingested in the morning as a liquid drink and in amounts representing 20 % of estimated 24 h food energy requirement all resulted in a rapid and strong reduction in circulating total and acylated ghrelin. Protein was the most potent. Lipids were the least potent. Interestingly carbohydrate ingestion resulted in a biphasic response; the ghrelin concentration decreased rapidly followed by a rebound increase in concentration. In all cases a change in insulin concentration could not explain the results.

Others also have reported that introduction of these nutrients individually into the stomach and duodenum, all directly stimulated a decrease in circulating ghrelin concentration [21, 22]. This suggests major nutrient sensing in these organs. However, controlling for merely a change in osmolality was not included in the protocol. In rats, merely increasing the osmolality in the upper intestine was sufficient to signal a suppression in ghrelin concentration [23].

Thus, the relative role of the various macronutrients in suppression of the ghrelin concentration and their possible interaction in mixed meals remains unclear.

Recently we designed a study to determine, in young, normal subjects, if the degree of saturation of fatty acids in dietary fats (lard, olive oil, safflower oil) affected the metabolic response to ingested carbohydrate (potato) [24]. In that study both total and acylated ghrelin responses were determined and were similar. It was a single meal study. The responses were modest, but all types of ingested fats resulted in a decrease in ghrelin, whereas the response to carbohydrate was biphasic. Thus, when these typical foods were digested, the responses were similar to those noted by Foster-Schubert et al. [10] when nutrient-specific beverages were ingested. The response did not correlate inversely and temporally with the glucose or insulin responses.

Based on data obtained in subjects without diabetes by Foster-Schubert et al. [10], an increased dietary protein, as well as fat, relative to the carbohydrate content, might be expected to decrease the ghrelin meal response. This is particularly so if protein, as observed by them, is most potent in suppressing ghrelin production. However, as indicated previously, in subjects with type 2 diabetes ingesting mixed meals, a greater decrease in ghrelin concentration was not present when the protein content was doubled [9]. Thus, the role of dietary protein in regulating the ghrelin concentration remains unclear.

Somewhat surprisingly, in the present study, the CHO-free, high fat diet resulted in a distinct, prolonged suppression of ghrelin throughout the day, i.e. the opposite of that expected based on the single meal studies in normal subjects [10, 13–17, 25, 26]. The reason for this is not entirely clear. These data, and the data of a dietary fat-induced decrease in ghrelin obtained previously in our single meal study in young, normal people [24], do indicate that dietary fat can result in a lower total ghrelin concentration either in the presence or absence of dietary carbohydrate and with no change in protein content in people with or without diabetes.

It should be noted that in our 5-week study cited above [9], an increase in dietary fat content from 30 % to 40 % and a decrease in CHO from 55 % to 30 % also did not affect the 24-h ghrelin concentration profile. Likewise, others reported that small changes in the carbohydrate:fat ratio [7] or the protein:fat ratio [27] in normal subjects did not affect the 24-hour profile.

Overall, the sensitivity to changes in fat content needs to be determined in subjects with and without diabetes and with and without simultaneous changes in protein and carbohydrate independently and over an 18–24 h period. If similar data are obtained in people without diabetes, it suggests that dietary fat is a major inhibitor of ghrelin secretion in general, although an absence of carbohydrate may make it more apparent.

In this regard, it also would be of interest to determine if a high fat, CHO-free diet-induced decrease in ghrelin also would inhibit appetite (food seeking). If so, would the effect be different in those with or without type 2 diabetes?

### Short-term starvation

In normal subjects, others have reported short-term starvation did not increase the overnight fasting concentration [12] or the 24-hour integrated ghrelin concentrations [28]. Striking circadian rhythms were reported to be present with a decreasing concentration during the day and an increase during the night [11].

In the present study in subjects with type 2 diabetes, 48 h without food also was associated with little change in the overnight morning concentration but with a very stable 24-hour ghrelin profile thereafter. Thus the absence of metabolic fuel intake per se does not result in an increase in ghrelin concentration as noted after weight loss (reviewed in [29]).

### Leptin

The overnight fasting leptin concentration is highly variable among people, but has been reported to be strongly associated with body fat mass [30, 31]. It also is affected by age and gender. It is higher in women, even if the higher fat mass in women is considered. It becomes lower with aging [30].

The circulating leptin concentration also was reported to have a striking circadian rhythm with the lowest levels at midday and a maximum at ~0200 h at night [32]. The rhythm is similar in young middle-aged normal men and women and in people with type 2 diabetes. This 24 h rhythm is entrained by the habitual meal timing of an individual [33]. However, in obese men and women, this occurred at a much higher leptin concentration [32, 34].

Studies in which the dietary macronutrient composition has been varied in weight-maintenance diets indicate little effect on the overnight fasting [35, 36] or 24 h leptin concentration [7, 27]. Leptin also does not respond acutely to meals [7, 27, 32].

Leptin is sensitive to changes in total food energy intake. This response is rapid and not dependent on weight loss (Reviewed in [37]). After an overnight fast, a decrease in concentration was demonstrated as rapidly as 15 h without food and was maximal at ~ 36 h. However, during a glucose infusion, which merely restored the overnight fasting concentration to that prior to the fast, there was no change in concentration even though the subjects were still grossly food-energy deficient, i.e. the leptin concentration did not decrease [38].

Also, in a study of obese subjects in which the morning leptin concentration was monitored daily during a 4 day period without food, the leptin had decreased to a low, stable level within 24 h. Interestingly, again infusion of a small amount of glucose, sufficient for a return of the glucose concentration to the level prior to the institution of the fast, resulted in a transient increase to that before food removal [31]. This occurred in the absence of a change in insulin concentration.

In the present study the 0800 h leptin had decreased 54 % at the end of the period without food. This decrease is similar to those noted by others in normal subjects [31, 38, 39] (Reviewed in [37]).

To our knowledge, the effect of an essentially CHO-free diet in people with or without type 2 diabetes has not been determined. The present data indicate that removing carbohydrate from a food energy-sufficient diet for 72 h results in a decrease in leptin concentration. The decrease was 35 % as much as with 72 h without macronutrient ingestion. The decrease in 24 h integrated glucose concentration was 70 % of that due to the absence of all food [1]. Thus, the current data in men with type 2 diabetes, as well as previous data in normal or obese individuals [31, 38, 40], suggest that the circulating glucose concentration [41] and/or maintenance of a defined overnight fasting glucose concentration can regulate the leptin concentration to food deprivation in general, either directly or perhaps indirectly [37].

Whether the current overnight fasting data reflect a 24-hour integrated concentration change remains to be determined. Quantitative 24-hour dose–response leptin data are necessary to fully assess the relationship of dietary glucose and/or maintenance of certain glucose concentrations on the leptin concentration dynamics.

## Conclusion

Current literature data indicate that there remains considerable uncertainty, both in people without and with type 2 diabetes, regarding the quantitative ghrelin response to specific macronutrients and/or their combinations. In the present study the 24-hr ghrelin dynamic response to meals when ingesting a typical mixed diet was similar to that reported in subjects without diabetes. However, the typical circadian rhythm was not apparent. Short-term starvation did not change the 24 h integrated total ghrelin concentration, but the meal-related changes were absent. Removal of carbohydrate from the diet and replacement with fat resulted in a daylong decrease in ghrelin concentration.

The overnight fasting leptin concentration was decreased 25 % by replacement of dietary carbohydrate with fat. It was decreased 55 % with short-term starvation.

## Abbreviations

CHO, carbohydrate; IV, intravenous; SDTU, special diagnostic & treatment unit
